# Investigating the interaction between white matter and brain state on tDCS-induced changes in brain network activity

**DOI:** 10.1016/j.brs.2021.08.004

**Published:** 2021

**Authors:** Danielle L. Kurtin, Ines R. Violante, Karl Zimmerman, Robert Leech, Adam Hampshire, Maneesh C. Patel, David W. Carmichael, David J. Sharp, Lucia M. Li

**Affiliations:** a Computational, Clinical, and Cognitive Neuroimaging Laboratory, Department of Medicine, Imperial College London, London, United Kingdom; b Neuromodulation Laboratory, School of Psychology, University of Surrey, Guildford, United Kingdom; c Centre for Neuroimaging Science, King's College London, Denmark Hill, London, United Kingdom; d Department of Biomedical Imaging, King's College London, 3rd Floor Lambeth Wing, St Thomas' Hospital, London SE1 7EH, United Kingdom; e Imperial UK Dementia Research Institute at Imperial Care Research and Technology Centre, United Kingdom

**Keywords:** Default mode network, Magnetic resonance imaging, Salience network, Stimulation, Traumatic brain injury, White matter structure

## Abstract

**Background:**

Transcranial direct current stimulation (tDCS) is a form of noninvasive brain stimulation whose potential as a cognitive therapy is hindered by our limited understanding of how participant and experimental factors influence its effects. Using functional MRI to study brain networks, we have previously shown in healthy controls that the physiological effects of tDCS are strongly influenced by brain state. We have additionally shown, in both healthy and traumatic brain injury (TBI) populations, that the behavioral effects of tDCS are positively correlated with white matter (WM) structure.

**Objectives:**

In this study we investigate how these two factors, WM structure and brain state, interact to shape the effect of tDCS on brain network activity.

**Methods:**

We applied anodal, cathodal and sham tDCS to the right inferior frontal gyrus (rIFG) of healthy (n = 22) and TBI participants (n = 34). We used the Choice Reaction Task (CRT) performance to manipulate brain state during tDCS. We acquired simultaneous fMRI to assess activity of cognitive brain networks and used Fractional Anisotropy (FA) as a measure of WM structure.

**Results:**

We find that the effects of tDCS on brain network activity in TBI participants are highly dependent on brain state, replicating findings from our previous healthy control study in a separate, patient cohort. We then show that WM structure further modulates the brain-state dependent effects of tDCS on brain network activity. These effects are not unidirectional - in the absence of task with anodal and cathodal tDCS, FA is positively correlated with brain activity in several regions of the default mode network. Conversely, with cathodal tDCS during CRT performance, FA is negatively correlated with brain activity in a salience network region.

**Conclusions:**

Our results show that experimental and participant factors interact to have unexpected effects on brain network activity, and that these effects are not fully predictable by studying the factors in isolation.

## Highlights

• We replicated the brain state and polarity dependent effects of tDCS.
• White matter structure influences tDCS's state-dependent changes in neural activity
• The parameters of tDCS may operate under a hierarchy of influence.

## Abbreviations

SN: salience network
FPCN: frontoparietal control network
DMN: default mode network
rIFG: right inferior frontal gyrus
dACC/pre-SMA: dorsal anterior cingulate/pre-supplementary motor area
PCC: posterior cingulate cortex
vmPFC: ventromedial prefrontal cortex

## 1 Introduction

Transcranial direct current stimulation (tDCS) is a powerful clinical and research tool that can improve cognition in healthy [1] and patient populations [2]. However, variability in the efficacy of tDCS, owing to our insufficient understanding and control of sources of intra and interindividual variability, is a hurdle to its widespread clinical application [3]. Participant factors, such as brain structure, and experimental factors, such as stimulation intensity, all influence tDCS's effects [3]. Multimodal neuroimaging facilitates the direct study of the physiological effects of stimulation. An increased understanding of how different factors influence the physiological effects of tDCS is invaluable to the rapidly developing field of tDCS for cognitive enhancement [[4], [5], [6]].

Brain state is a key factor determining the physiological response to tDCS. Previous work from our group and others have found that brain state influences the brain network effects of tDCS in healthy controls [[7], [8], [9]]. Specifically, our previous tDCS-fMRI work in healthy controls shows that tDCS reinforces the underlying pattern of brain network activity, and that this interacts with stimulation polarity [9]. However, we have not investigated the interaction between brain state, stimulation polarity and WM structure.

Work from our group [10,11] and others [12] have found that WM structure is a key factor in determining the behavioral response to tDCS. Traumatic brain injury (TBI) characteristically results in traumatic axonal injury due to axonal shearing, where forces tear delicate WM axons [13,14]. This makes TBI a useful model of reduced white matter connectivity. We have previously found that higher WM structural connectivity, as assessed by fractional anisotropy with Diffusion Weighted Imaging, is related to greater behavioral improvements in a cognitive control task in both healthy and TBI participants [10,11]. However, there is no previous study investigating the interaction of multiple factors on brain network activity.

Using a cohort of healthy control and TBI participants partially described in previous publications [9,11], we were able to investigate the interactive effects of brain state, stimulation polarity, and WM structure on the brain network effects of tDCS. By replicating some of our previous methods used in a healthy control group in a group of patients with TBI, we hope to address the variability among tDCS studies [3], as well as the ‘reproducibilty crisis’ afflicting many fields, including biological sciences [15].

We used the Choice Reaction Task (CRT) to manipulate brain state. The CRT is a well-described task that produces robust patterns of anti-correlated activity in the salience network (SN) and default mode network (DMN) in TBI patients and healthy controls [16]. Additionally, the CRT is a simple cognitive task with which both TBI patients and healthy controls can engage with high (>90.0%) levels of accuracy [16], producing the core pattern of SN activation and DMN deactivation in both cohorts. In our previous work, stimulation did not improve behavioral performance in healthy participants [9], enabling changes in brain network activity to be interpreted independently of changes in performance.

We targeted anodal, cathodal, and sham stimulation to the right inferior frontal gyrus (rIFG) during execution of the CRT and “rest” blocks. The rIFG is a control node for DMN/SN dynamics [[17], [18], [19], [20], [21]], mediating the switch from the DMN to a task-active state [18,19].

In line with our previous findings in healthy controls that tDCS accentuates the brain network activity associated with the underlying brain state, we hypothesized that brain state is a core determinant of tDCS's effects on brain networks in TBI patients. We further hypothesize that WM structure will additionally modulate the physiological effects of tDCS, with higher WM structural connectivity corresponding to greater brain network activity.

## 2 Methods

### 2.1 Participants

We recruited 58 participants, consisting of healthy controls (n = 24; 12F:12 M; mean age = 39 years, SD = 15.8 years) and TBI patients (n = 34; 5F:29 M; mean age = 39.4 years, SD = 10.1 years) (Table 1). Clinical characteristics of this cohort are previously described [11] and summarized in Table 1. Extensive clinical data, including MR findings, can be found in Supplementary Information Table 1. Inclusion and exclusion criteria are described in Table 2. One TBI participant was excluded due to distortion in fMRI preprocessing. Diffusion weighted MRI was collected from twenty-two healthy controls and thirty-three TBI patients. Data from a second experiment, performed on the same day after this experiment, on the same group of control and TBI participants, examining the impact of white matter on behavioral performance has been previously published [11]. All participants were naïve to tDCS and gave written informed consent. Ethical approval for the study was granted through the local ethics board (NRES Committee London-West London and GTAC). Post-hoc power analyses confirmed we were adequately powered (≥0.80) to address the aims of this study (Supplementary Information Post Hoc Power Analysis).

**Table 1:** TBI patient and healthy control demographic information.

	TBI Patients	Healthy Controls
n	n = 34	n = 24
Gender (M:F)	30:5	12:12
Age (years ± SD)	39.7 ± 10.0	39.0 ± 15.8
Age at injury (years ± SD)	32.8 ± 11.5	n/a
Time since injury (months ± SD)	93.5 ± 94.3	n/a
Lesion volume (%)	0.6 ± 1.3	n/a

**Table 2:** Participant inclusion and exclusion criteria.

**Exclusion Criteria**	**Inclusion Criteria**
Significant premorbid neurological or psychiatric illness, or learning disability	A diagnosis of moderate-severe TBI (based on Mayo Clinic classification)
Substantial lesion directly affecting the right inferior frontal gyrus	Able to provide informed consent for participation
Previous head injury	Aged 16–80 years old
Contraindication to tDCS (seizures within 12 months, broken skin over the site of stimulation, metallic implants in skull or eyes)	Subjective or objective evidence of cognitive impairment after TBIAt least 6 months since time of injury
Cognitive impairment of such severity that the subject is unable to cooperate with the study	Clinically stable following TB
Contraindication to MRI scanning (including pregnancy)	Naïve to tDCS Able to complete scanning protocol
Significant premorbid neurological or psychiatric illness, or learning disability	A diagnosis of moderate-severe TBI (based on Mayo Clinic classification)
Substantial lesion directly affecting the right inferior frontal gyrus	Able to provide informed consent for participation
Previous head injury	Aged 16–80 years old
Contraindication to tDCS (seizures within 12 months, broken skin over the site of stimulation, metallic implants in skull or eyes)	Subjective or objective evidence of cognitive impairment after TBI
Cognitive impairment of such severity that the subject is unable to cooperate with the study	Clinically stable following TBI
Contraindication to MRI scanning (including pregnancy	At least 6 months since time of injury Able to complete scanning protocol

### 2.2 tDCS-fMRI paradigm

**Fig. 1:**
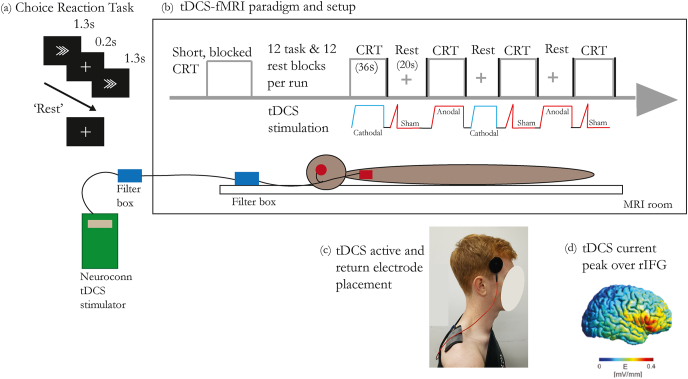
(a) Stimuli of the CRT and (b) experiment paradigm with concurrent tDCS/fMRI. Each participant performed three runs, each run consisting of four blocks of the six unique combined stimulation and task conditions (CRT + anodal, CRT + cathodal, CRT + sham, “rest” + anodal, “rest” + cathodal, “rest” + sham). Blocks were interspersed by a blank screen with no stimulation. (c) Photo of the tDCS experimental setup. (d) Modelling of the electric field distribution over the rIFG.

The Choice Reaction Task (CRT) consists of a random sequence of left or right arrows, to which participants had 1.3 s to respond by pressing a button with the index finger of the corresponding hand (Fig. 1a). The tDCS-fMRI paradigm consisted of alternating blocks of CRT and “rest”. During the task and rest blocks participants received anodal, cathodal, or sham tDCS, creating six different conditions of combined brain state and stimulation polarity: “rest”+sham; “rest”+anodal; “rest”+cathodal; CRT + sham; CRT + anodal; CRT + cathodal (Fig. 1b). Each run comprised 4 blocks of each condition, interspersed with brief black screen periods, presented in the same, pseudo-randomized order for all participants. Within each run, there were 12 “rest” blocks (20 s each) and 12 task blocks (34 s each). Participants performed three runs interspersed with 2–3 min of rest to prevent fatigue. This resulted in a total of 18 min of full intensity tDCS, 12 min of “rest”, and 20 min, 24 s of task. Prior to the tDCS-fMRI paradigm, participants performed a shorter version of the CRT. This was done to create masks for the region of interest analysis evaluating the interaction between the brain state and polarity dependent on stimulation-induced brain network activity, and confirm the task produced the expected patterns of brain network activity in our population. If stimulation during the CRT produced unexpected changes in neural network patterns, we felt it prudent to include a CRT without stimulation, to compare and confirm that the changes were not a result of the task itself.

### 2.3 Delivery of tDCS

The active electrode was placed at F8 (10–20 EEG system) and the return electrode on the right shoulder (Fig. 1c). Pre-stimulation impedances were below 3 kOhm, and maximum impedance during stimulation was 29 kOhm. To reduce impedance, electrodes were affixed with a layer of conductive paste (Ten20, D.O. Weaver, Aurora, CO). Anodal and cathodal stimulation were delivered using a 4.5 s ramp up, followed by full stimulation intensity at 1.8 mA, then a 0.5 s ramp-down (Fig. 1b). Sham stimulation consisted of ramp stages only. TTL triggers from the scanner were sent to the main computer, which controlled the simulation triggers via National Instruments DAQ device (National Instruments, Newbury, UK) using in-house MATLAB scripts. RF filtering boxes were located both inside the control room and the scanner bore and connected by Y-cable (RF-59/ULF; length = 3 m) and custom-made filter in the penetration panel to the stimulator in the control room. Wires from the participant and scanner were routed via the back of the bore to the control room. Tests used to ensure safe operating standards were conducted as per [22]. Peak electric field was confirmed via computational, Finite Element Method (FEM) head modelling as in Li et al. [9] (Fig. 1d). Outside the scanner, participants had two 15-s blocks of anodal and cathodal tDCS, and were asked to rate sensations of itching, pain, metallic taste, burning, anxiety, and tingling on a 1–5 scale, with 1 = nil and 5 = unbearable. The average ratings for all categories found no difference between anodal or cathodal tDCS and participants were unable to determine which stimulation condition they experienced. Further details of the experimental paradigm and stimulation can be found in Ref. [9].

### 2.4 Non-diffusion structural and functional MRI acquisition and preprocessing

T1 and fMRI sequences were acquired using a 3 T S Verio (Siemens, Erlangen, Germany) and 32 channel head coil using parameters modeled from Ref. [22]. fMRI data preprocessing was performed as in Ref. [9] using FMRI Expert Analysis Tool (FEAT) Version 6.00, from FMRIB's Software Library (FSL) [23]. This carried out brain extraction to remove non-brain tissue (BET) [25] and motion correction, performed using MCFLIRT [24]. FEAT was used to conduct removal of low-frequency drifts, spatial smoothing used a Gaussian kernel filter with a full width at half maximum of 6 mm. Using the participant's T1-weighted scan and preprocessed data, participant's fMRI volumes were registered to Montreal Neurological Institute (MNI) 152 standard space. Independent component analysis (ICA) analysis was conducted on each run using Multivariate Exploratory Linear Optimized Decomposition (MELODIC) [26]. Components were classified using a trained, automatic classification software, FMRIB's ICA-based Xnoisefier (FIX) [27]. Each ICA component was manually checked, and components considered to be artefact, motion or otherwise, based on criteria from Ref. [28] were removed. This was done for every participant in both groups.

### 2.5 fMRI analysis: activation

A general linear model (GLM) was used to determine the relationship between activation and the task or “rest” conditions. In brief, single-session, subject level GLMs were first conducted to determine the effects of anodal and cathodal stimulation during the task, with the following regressors of interest: [all task blocks], [all anodal blocks], [all cathodal blocks]. The interactive regressors [task + anodal] and [task + cathodal] were run to demonstrate the interactive effects of stimulation polarity (anodal or cathodal) and brain state. The implicit baseline consisted of the [“rest”+sham] blocks and was not included in the GLM. Subject levels were run again using [all rest block] rather than the [all task block] to demonstrate the effects of anodal and cathodal tDCS during the resting state. To interrogate the effects of stimulation in the absence of task a second GLM was constructed, using “rest” for task, resulting in [all “rest” blocks]. The interactive regressors [“rest”+anodal] and [“rest”+cathodal] then demonstrated the interactive effects of anodal and cathodal stimulation in the absence of task. All were run using square wave, double-gamma HRF in FSL's FEAT. Six movement regressors were included in first-level FEAT analysis as a covariate of no interest, to account for motion artefacts. A regressor of no interest to account for the periods of black screen was included. Group-level, mixed effects analysis combined all participant's sessions, then all participants were combined using FLAME 1 + 2 in FSL FEAT. The parameter estimates [task + anodal], [task + cathodal], [“rest”+anodal], [“rest”+cathodal], [“rest”+anodal] >[“rest”+cathodal], and [task + anodal] >[task + cathodal] were run, as were the inverse estimates. Z statistical images were thresholded using Gaussian random field-based cluster inference with threshold of Z > 3.1, corrected for cluster significance at a threshold of p = 0.05. Further details can be found in Ref. [9]. Determining the interaction between state and polarity dependent effects of tDCS on TBI patients was conducted with a region of interest (ROI) approach. The “task activated” and “task deactivated” network consisted of a binarized mask of the regions of increased (task activated) or decreased (task deactivated) BOLD activation during the shorter, non-stimulated, blocked CRT during the [task>“rest”] contrast.

### 2.6 Diffusion tensor imaging (DTI) acquisition and analysis

Diffusion weighted imaging was collected from n = 22 healthy control and n = 33 TBI participants. Diffusion-weighted volumes were acquired using a 64-direction protocol (64 slices, in-plane resolution = 2 × 2 mm, slice thickness = 2 mm, field of view = 25:6x25:6 cm, matrix size = 128x128, TR = 9500 ms, TE = 103 ms, b-value = 1000 s/mm^2^). Four images were also acquired without diffusion weighting (b-value = 0 s/mm^2^). DTI data were corrected for head motion and eddy current distortions, brain mask was extracted and constrained the tensor model using FSL's FMRIB's Diffusion Toolbox. Application of the tensor model generated voxelwise, individual FA maps. The maps were transformed into 1 mm-resolution standard space using DTI-TK [29]. An initial group-based template was generated [30], and individual tensor-based images were then registered to the group template using diffeomorphic transformations. To assess WM structural connectivity in the whole brain and of regions composing the two brain networks of interest (SN and DMN) FA values were extracted for the following structures as obtained in Ref. [31]:

1. The Whole Skeleton tract assesses whole-brain WM tract integrity.
2. The rAI-dACC/pre-SMA tract assesses SN structural integrity in the tract connecting the right anterior insula (rAI) to the dorsal anterior cingulate (dACC)/pre-supplementary motor area (pre-SMA). This tract partly overlaps the frontal Aslant tract described by Ref. [31].
3. The mPFC-PCC/PRE (medial prefrontal cortex to posterior cingulate cortex/precuneus) tract assessed DMN structural integrity within the bilateral cingulum.

The influence of white matter structure on the brain state and polarity dependent effects of stimulation were determined using FA as a continuous covariate, in higher-level FEAT analyses as single-group averages with continuous covariate interaction. Parameter Estimate values were extracted from the areas of significance to demonstrate the directionality of the relationship between the physiological effects of stimulation and WM structure.

DVARS assess the impact of motion on the BOLD signal by using backward differentiation to compute the root mean squared change in intensity between frames of an fMRI timeseries [32]. To test whether motion outliers confounded the analysis of white matter structure on tDCS-induced changes in brain activity the average DVARS per subject per session were computed. The influence of motion on the brain state and polarity dependent effects of stimulation were determined using DVARS values as a continuous covariate in higher-level FEAT analyses as single-group averages with continuous covariate interaction.

### 2.7 Statistical analysis of behavioral results

Statistical analysis was conducted using MATLAB 2019 [33] and R (www.r-project.org). Relationships between unequally sized groups were determined using randomization tests run for 1000 permutations.

## 3 Results

### 3.1 The brain network effects of TDCS are dependent on cognitive brain state in both healthy and TBI participants

**Fig. 2:**
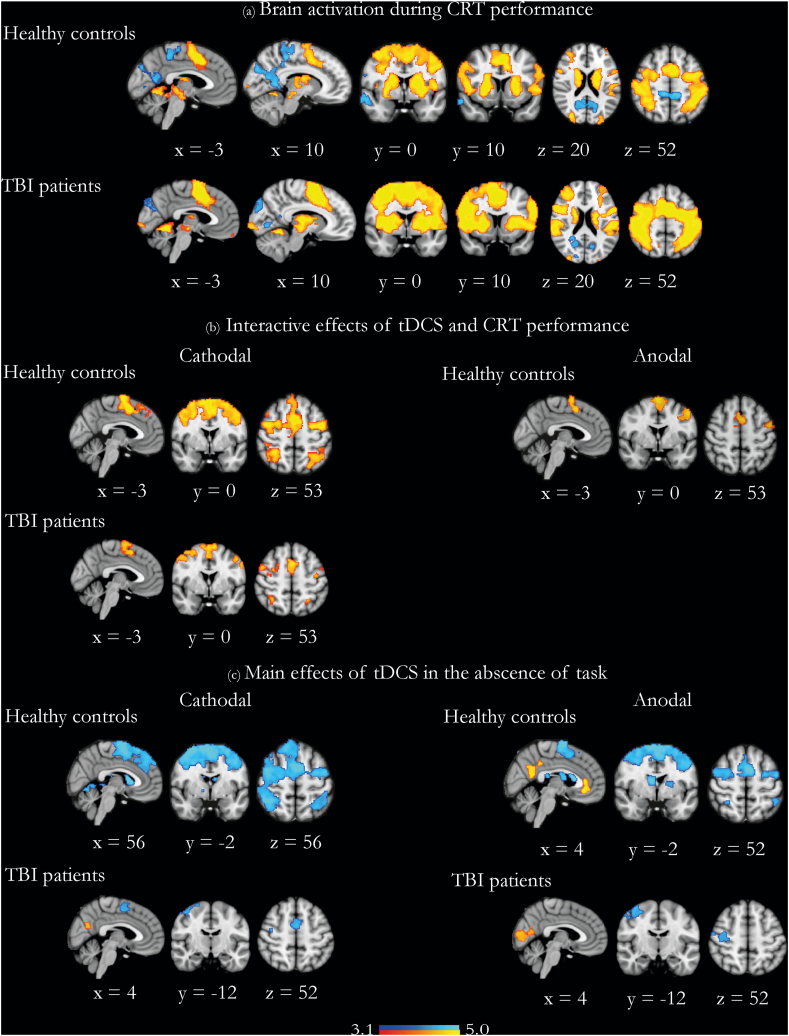
The physiological effects of tDCS during and in the absence of the Choice Reaction Task. (a) Overlay of brain activation (in warm colors) and deactivation (in cool colors) during the short CRT with no tDCS, in healthy and TBI participants. (b) Brain regions with further activation due to anodal and cathodal stimulation applied during CRT performance in healthy participants, and brain regions with further activation due to cathodal stimulation applied during CRT performance in TBI participants. (c) Brain regions showing the main effect of anodal and cathodal stimulation in the absence of task in healthy and TBI participants. Warm colors denote further activation, and cool colors denote further deactivation. Results are superimposed on a 1 mm MNI152 template. Note that the healthy control results have been previously described, with full analysis presented in Li et al., 2018 [9] and are presented here to provide context for interpretation of the results from the TBI cohort. Cluster corrected z = 3.1, p < 0.05. (For interpretation of the references to color in this figure legend, the reader is referred to the Web version of this article).

The findings in the TBI cohort almost entirely replicate our previous work in healthy participants [9]. Indeed, there were no whole-brain differences when contrasting TBI and healthy participants in any of the five conditions presented in Fig. 2.

In both healthy and TBI participants, CRT performance without stimulation displayed robust activation across fronto-parietal regions, including the dACC node of the SN, primary sensory/motor cortex, basal ganglia, and bilateral thalami (Fig. 2a). There was concurrent deactivation of the posterior cingulate cortex (PCC) node of the DMN.

The interactive effects of tDCS and CRT performance and the main effect of stimulation in the absence of task have been previously described in this control cohort [9]. In essence, both anodal and cathodal stimulation act to accentuate the underlying patterns of brain network activity set by the current cognitive brain state. That is, the interactive effect of anodal or cathodal tDCS and CRT performance was additional increased activation in the dACC/pre-SMA and lateral prefrontal regions, which are all areas already activated during CRT performance (Fig. 2b). Conversely, the main effect of anodal and cathodal tDCS in the absence of task was increased deactivation of the rIFG and underlying anterior insula, bilateral superior frontal gyri, bilateral frontal eye fields, and bilateral superior parietal regions, areas which are already active in the absence of task. An additional main effect of anodal tDCS in the absence of task was further activation of the PCC, the precuneus, and the ventromedial prefrontal cortex (vmPFC), areas associated with the DMN (Fig. 2c).

In TBI participants, the interactive effect of cathodal tDCS and CRT performance was additional increased activation in the dACC node of the SN and, to a lesser extent, primary sensory/motor cortex, basal ganglia, and bilateral thalami, which are all areas already activated during CRT performance (Fig. 2b). There was no interactive effect of anodal tDCS and CRT performance over and above the brain activity patterns elicited by CRT performance itself. Conversely, the main effect of cathodal tDCS in the absence of task was increased activation in PCC and further deactivation of dACC, and the main effect of anodal tDCS in the absence of task was increased activation in medial occipital cortex and further deactivation of sensory/motor cortex (Fig. 2c).

### 3.2 The brain state-dependent effects of tDCS in TBI patients are different between anodal and cathodal stimulation

An ROI approach was used to investigate the interaction between the brain state and polarity dependent effects of tDCS [9], focusing the analysis to brain network areas relevant to CRT performance. Using linear mixed effects modelling, we found a significant interaction between brain state and stimulation polarity (F[1263] = 8.74, p = 0.003). This interaction holds when analyzed for both the task-activated regions (F[1213] = 4.23, p = 0.042, η^2^ = 0.031) and task-deactivated regions (F[1412] = 4.16, p = 0.043, η^2^ = 0.030).

During both task and fixation, both stimulation polarities modulated BOLD activity to emphasize underlying network activity. Post-hoc randomization tests confirmed cathodal stimulation during task produced greater BOLD activity than activation due to cathodal stimulation in the absence of task (p = 0.00008, d = 0.65). Similarly, cathodal stimulation during task produced greater BOLD activity than activation in the absence of task due to anodal stimulation p = 0.05, d = 0.49). In the absence of task, activity due to cathodal stimulation was less pronounced than anodal stimulation during task (p = 0.004, d = −0.47). There was no difference between the effects of anodal and cathodal stimulation in the task-activated regions. Overall, the marked effects seen in cathodal stimulation were not seen with anodal stimulation.

### 3.3 WM structure further modulates the brain state-dependent effects of tDCS on brain network activity

**Fig. 3:**
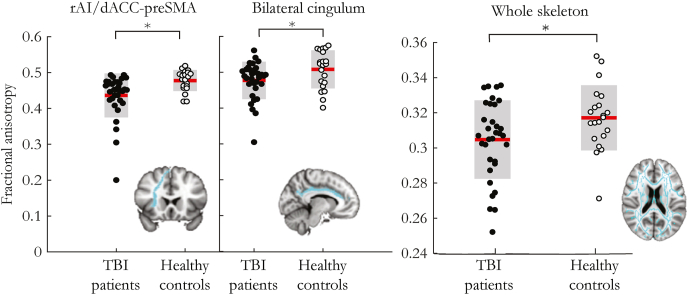
WM structure in key tracts in TBI patients and healthy controls. Red lines are group mean values. Grey boxes are ±1 standard deviation from the mean. Inset brain pictures show rAI-dACC/pre-SMA, bilateral cingulum and whole skeleton tracts. ∗ denotes p < 0.05. (For interpretation of the references to color in this figure legend, the reader is referred to the Web version of this article.)

**Fig. 4:**
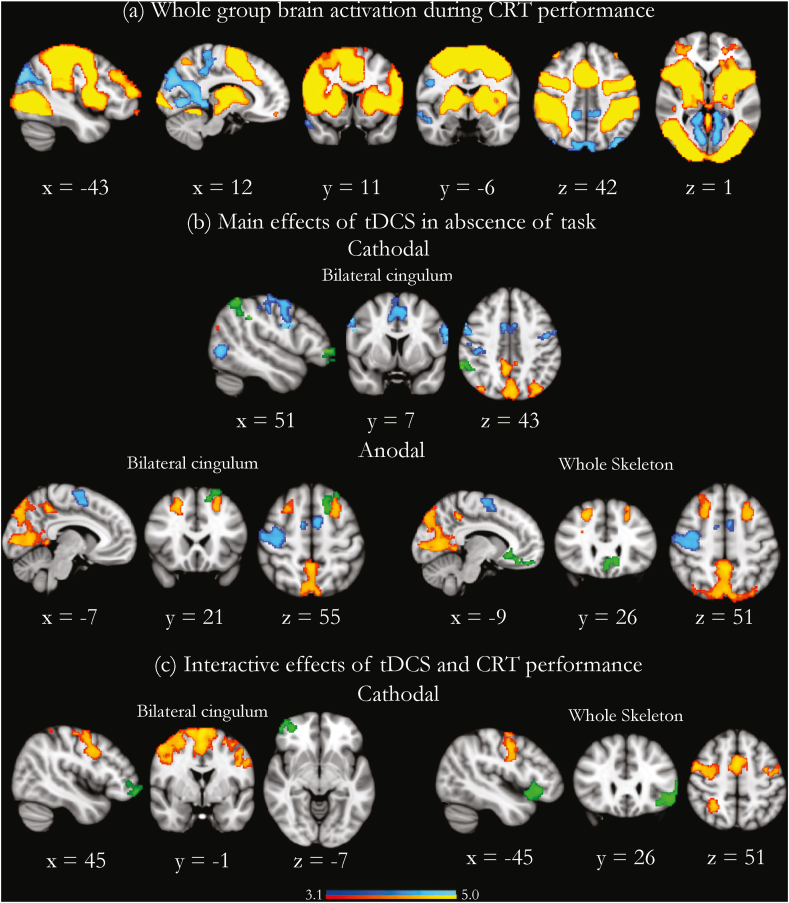
The influence of WM structure on physiological effects of tDCS during and in the absence of task. (a) Overlay of brain activation (in warm colors) and deactivation (in cool colors) during CRT performance in the whole group (TBI patients and healthy controls. b) Brain regions where WM structure further modulates stimulation-induced BOLD activity in the absence of task (in green). Warm and cool colors denote the BOLD activity produced by stimulation, without accounting for white matter structure. (c) Brain regions where WM structure further modulates stimulation-induced BOLD activity during task performance (in green). Warm and cool colors denote the BOLD activity produced by stimulation, without accounting for white matter structure. Results are superimposed on a 1 mm MNI152 template. Cluster corrected z = 3.1, p < 0.05. (For interpretation of the references to color in this figure legend, the reader is referred to the Web version of this article.)

Randomization tests showed there was a main effect of group on FA (p = 0.0001, *d* = −0.9). Post-hoc randomization tests showed TBI participants had reduced mean FA within the whole skeleton (p = 0.02, *d* = −0.58) in the rAI-dACC/pre-SMA tract representing the SN (p = 0.001*, d* = −1.9) and in the cingulum bundle, representing the DMN (p = 0.001*, d* = −0.74) (Fig. 3). As there were no group differences in the brain-state dependent effects of tDCS, TBI patients and healthy controls were combined to determine the influence of WM structure on the physiological effects of stimulation (Fig. 4a).

**Fig. 5:**
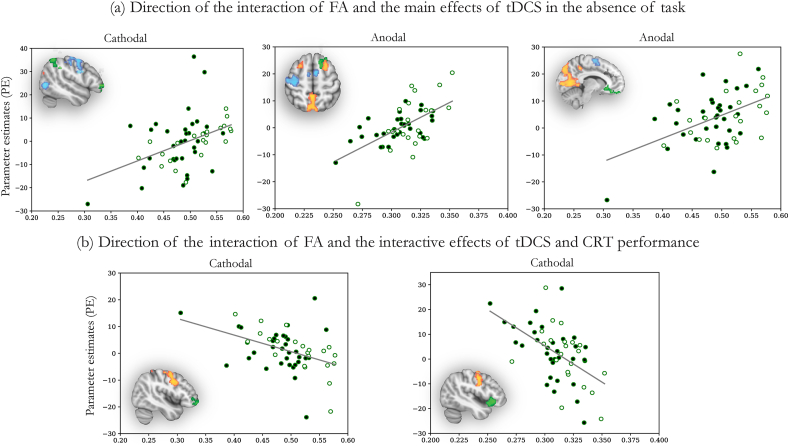
The direction of the interaction of the influence of WM structure on physiological effects of tDCS during and in the absence of task. Scatter plots demonstrate the nature of the relationship between WM structure and stimulation-induced BOLD activity. (a) Direction between FA and the main effect of tDCS in the absence of task for TBI patients (black circles) and healthy controls (white circles). BOLD activity was extracted from the green regions in the brain insets, which correspond to the results shown in Fig. 5b. (b) Direction between FA and the interactive effects of cathodal tDCS and CRT performance in TBI for TBI patients (black circles) and healthy controls (white circles). BOLD activity was extracted from the green regions in the brain insets, which correspond to the results shown in Fig. 5c. For all plots, FA is on the x-axis, and parameter estimates indicate the level of BOLD activity on the y-axis. Line of best fit is fitted to the whole-group data to highlight the direction of influence. (For interpretation of the references to color in this figure legend, the reader is referred to the Web version of this article.)

*In the Absence of Task:* Bilateral cingulum FA influenced BOLD response within several regions to cathodal stimulation: the right frontal pole, middle frontal gyrus, and inferior parietal lobule (Fig. 4b). Increases in bilateral cingulum FA correlated with increases in activity in these regions (r_s_ = 0.446, p = 8.25 e−04) (Fig. 5a). Whole skeleton FA modulated BOLD response within the vmPFC during anodal stimulation in the absence of task (Fig. 4b). As whole skeleton FA increases, vmPFC activation increases (r_s_ = 0.500, p = 1.44 e−04) (Fig. 5a). Bilateral cingulum FA influenced BOLD response within the left middle frontal gyri to anodal stimulation in the absence of task (Fig. 4b). As bilateral cingulum FA increases, so does activation in the left middle frontal gyri (r_s_ = 0.361, p = 0.008) (Fig. 5a). In summary, there were positive correlations between WM structure and brain network activation in the absence of task. There was no influence of whole skeleton FA on brain activity due to cathodal stimulation, and there was no influence of rAI-dACC/preSMA FA on brain activity due to anodal or cathodal stimulation. There was no influence of motion (as measured via DVARS) on the BOLD response on any of the regions where white matter structure influenced tDCS-induced changes in brain activity.

*During Task Performance:* Whole skeleton FA influenced the BOLD response within the left IFG with cathodal stimulation applied during task performance (Fig. 4c). As whole skeleton FA increased, there was more left IFG deactivation (r_s_ = 0.433, p = 0.001) (Fig. 5b). Bilateral cingulum FA influenced the BOLD response within the right IFG/right frontal pole with cathodal stimulation applied during task. As bilateral cingulum FA increases, there was more right IFG/frontal pole deactivation (r_s_ = 0.433, p = 0.001) (Fig. 5b). In summary, there was an inverse relationship between WM structure and brain network activity with tDCS applied during task performance. There was no influence of rAI-dACC/preSMA FA on brain activity during cathodal stimulation. There was no influence of FA from any of the tracts on brain activity during anodal stimulation. There was no influence of motion (as measured via DVARS) on the BOLD response on any of the regions where white matter structure influenced tDCS-induced changes in brain activity.

### 3.4 Behavioral performance in the Choice Reaction Task

**Fig. 6:**
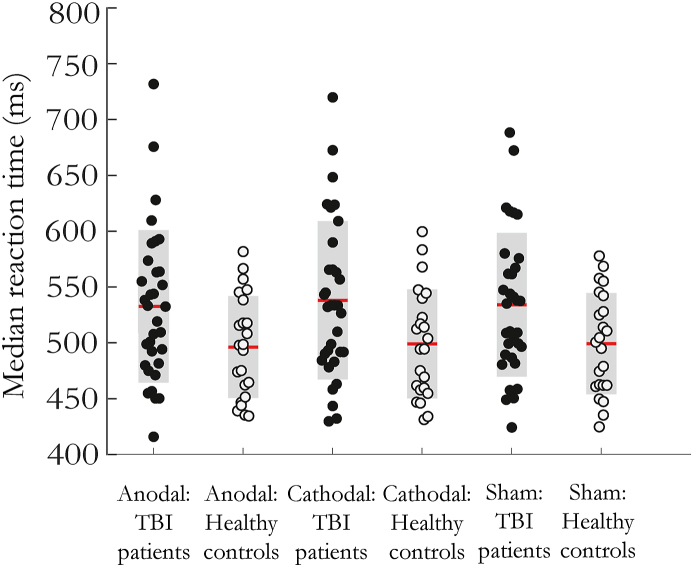
Median reaction time on the CRT in healthy controls (open circles) and TBI patients (filled circles) with anodal, cathodal, and sham stimulation. Red lines are group mean values. Grey boxes are ±1 standard deviation from the mean. (For interpretation of the references to color in this figure legend, the reader is referred to the Web version of this article.)

There was a main effect of group on median reaction time (F[1,13.57] = 168, p = 0.0003, η^2^ = 0.11), with TBI participants showing slower reaction times than healthy participants. There was no main effect of group on accuracy (F[1,2.556] = 171, p = 0.112, η^2^ = 0.037), with both groups performing at >90% accuracy in all conditions (Fig. 6). There was no main effect of stimulation type or interactions between group and stimulation type on reaction times or accuracy (all p > 0.05).

## 4 Discussion

We show that brain state is a key factor that determines the physiological effects of tDCS in TBI participants. This replicates findings from our previous study in a separate, clinical cohort. Replication studies are of particular importance in the field, give the variability of results. We further show that WM structure modulates the influence of brain state on brain network effects of tDCS. In a field with a vast parameter space, our results show how participant and experimental factors interact to shape the physiological effects of tDCS. This introduces the idea of a ‘hierarchy of influence’ within the parameter space, and extends our mechanistic understanding of tDCS's effect on brain network dynamics.

### 4.1 Replicating the brain state dependent effects of tDCS

We have previously shown that brain network effects of tDCS are highly dependent on underlying cognitive brain state [9]. We now replicate this finding in a new patient cohort, which suggests that the brain-state dependence of tDCS action is a core characteristic of tDCS-induced neural activity. As we've shown in healthy controls, tDCS seems to reinforce underlying brain network activity. During the CRT, cathodal stimulation increases SN activity over and above endogenous activity. In the absence of task, the same stimulation polarity actually produced decreased SN activity, thus reinforcing state-dependent patterns of brain network activity. We have shown the state-dependent effects of tDCS in both healthy control [9] and now a patient population. This suggests that brain state is a particularly important determinant of tDCS's effects and is broadly generalizable across populations. This framework seems to generalize to other modalities [34,35]. For example, a study investigating tDCS-induced changes in cortical excitability, as assessed by motor evoked potentials, found the physiological effects of tDCS were dependent on brain state [36]. This diverges from the anodal-excitatory, cathodal-inhibitory dichotomy seen on a cellular level [36]. It has been hypothesized that the different effects on the macro/meso level compared to the microscopic level could result from tDCS's effects on a cellular level operating on various excitatory and inhibitory populations, shifting the balance of large-scale, state-dependent brain networks [37].

### 4.2 The brain state-dependent effects of tDCS on brain network activity interact with WM structure

We found that WM structure influences tDCS-induced brain activity in a state-dependent manner. In the absence of task, higher FA was correlated with increased brain activity in vmPFC, rIFG, middle frontal gyrus, and IPL. These areas, particularly IPL and vmPFC, are part of the DMN, and usually show increased activity in the absence of CRT performance for both anodal and cathodal stimulation. We have already shown that the effects of tDCS are brain state dependent, and now find that, in the absence of task, WM structure seems to accentuate the state-dependent response to tDCS.

One possible reason for the state-dependent directionality of WM structure's influence on brain network activity might be the extent of structural connectivity to the node itself, and its functional role in varying brain network dynamics. In the absence of task, we found bilateral cingulum FA influenced increased activity in the IPL, left middle and frontal gyri. The IPL is a component of the DMN and FPCN, and has been identified for its role in attentional control [38]. This explains why bilateral cingulum FA influences increased activation of the IPL-a region with high functional and structural overlap with the DMN and SN. A recent computational simulation study investigated the influence of structural and functional connectivity of a node's ability to change brain network activity upon stimulation [39]. They found that stimulating the IPL drives brain network activity to a target state, due partially to its high structural connectivity [39,40]. This strengthens the theory that in the absence of task WM structure influences the brain state-dependent physiological effects of tDCS.

Conversely, during task performance, there was a negative relationship between FA and brain network activity, seen in bilateral IFGs. These are regions which, in our population, show an increase in activity during CRT performance. We have previously shown that healthy controls in a high FA group demonstrated greater rIFG and right anterior insula activation than a low FA group during anodal tDCS during a different, more complex cognitive task [10]. This led us to predict that higher FA would result in increased rIFG/left IFG activation with tDCS during CRT performance. This unexpected finding suggests that during CRT performance the relationship between WM structure and tDCS-induced brain activity is no longer injective. One possible extension of this study would be to vary the degree of task difficulty as a way to continuously manipulate brain state, to map out the interaction between WM structure and brain state in more detail.

Therefore, we see that the same stimulation polarity (cathodal) and same WM tract (bilateral cingulum) seem to shape the brain network effects of stimulation in different ways depending on brain state. This leads us to speculate that there is a ‘hierarchy of influences’ on brain network activity response to tDCS, where some factors are more influential than others in determining brain network response to tDCS. Future research could utilize computational models to further investigate the influences and interactions of the parameters of stimulation. For example, Giannakakis et al. used a series of connected Wilson Cowan oscillators to investigate how inter-regional connectivity and excitability of stimulated regions influenced the long-term effects of stimulation [41] and Muldoon et al. used a similar system to investigate how between-subject variability, structural, and functional connectivity influence the brain network effects of stimulation [42].

### 4.3 Limitations

Our stimulation target was limited to the rIFG. Though we chose the rIFG for its unique role in the SN/DMN dynamics, we postulate stimulation of other nodes in the DMN or SN- such as the IPL-would encourage similar network driven results. There was a higher proportion of males in the TBI cohort compared to the healthy control group. There is evidence that sex influences the physiological effects of tDCS [43]. There was no difference between our groups in their brain network responses to tDCS, so we are not able to comment further on the influence of sex on the interactions between tDCS parameters. However, this sex disparity should be considered when attempting to extrapolate the results of healthy control tDCS studies to the TBI patient population. The pseudorandomized nature of our study was a precaution against carry-over effects of stimulation. If there is some carryover, the pseudorandomisation is more likely to produce noise rather than systematic bias. Furthermore, our fMRI analysis ensures carryover effects would more likely result in a false negative result than a false positive by investigating the influence of tDCS on brain network activity during either [task + stimulation] or [“rest” + stimulation] over and above [sham + task] and [sham + “rest”]. Furthermore, the motor literature suggests that 3 min of stimulation in the motor cortex was required to induce after effects [44]. Our task had maximum of 34 s of continuous tDCS, far below the threshold needed to produce after effects.

## 5 Conclusion

We provide further evidence for the important influence of brain state on the brain network effect of tDCS. We further show that WM structure interacts with brain state in its effect on tDCS-induced brain network activity. It is possible that participant and experimental factors follow a ‘hierarchy of influence’, with factors such as brain state exerting a greater, more generalizable influence on the physiological effects of tDCS because it is higher up on the ‘hierarchy’. Further studies specifically investigating parameter interactions on tDCS-induced brain network activity will help improve our ability to translate the use of stimulation.

## Funding

This work had the following funding: LML was funded by 10.13039/100010269Wellcome Trust Clinical Research Training Fellowship (103429/Z/13/Z) and the 10.13039/501100000272NIHR Academic Clinical Lectureship program. I.R.V. was funded by the 10.13039/100010269Wellcome Trust (Ref: 103045/Z/13/Z) and the 10.13039/501100000268BBSRC (Ref: BB/S008314/1), DJS is funded by a 10.13039/501100000272National Institute for Health Research (10.13039/501100000272NIHR) Professorship.

## CRediT authorship contribution statement

**Danielle L. Kurtin:** Formal analysis, Investigation, Data curation, Writing – original draft, Visualization. **Ines R. Violante:** Conceptualization, Methodology, Software, Validation, Supervision, Project administration, Writing – review & editing. **Karl Zimmerman:** Validation, Investigation, Project administration, Writing – review & editing. **Robert Leech:** Methodology, Writing – review & editing. **Adam Hampshire:** Methodology, Writing – review & editing. **Maneesh C. Patel:** Formal analysis. **David W. Carmichael:** Methodology, Validation. **David J. Sharp:** Conceptualization, Methodology, Resources, Data curation, Supervision, Project administration, Funding acquisition, Writing – review & editing, Supervision, Resources. **Lucia M. Li:** Conceptualization, Methodology, Software, Validation, Investigation, Formal analysis, Resources, Data curation, Supervision, Project administration, Funding acquisition, Writing – review & editing.

## Declaration of competing interest

The authors declare there are no known conflicts of interest associated with this publication.
