# Supplementary material for: Investigating the interaction between white matter and brain state on tDCS-induced changes in brain network activity
Source: Brain Stimul. 2021 Sep-Oct;14(5):1261–70. doi: 10.1016/j.brs.2021.08.004 (PMC8460997; doi:10.1016/j.brs.2021.08.004)
Supplement: Multimedia component 1 [file mmc1.docx]

**SUPPLEMENTARY INFORMATION:**

**Post-hoc Power Analysis:**

To determine if our sample size provided adequate power (≥ .80), we conducted post-hoc power analysis using G*Power 3. We set α = 0.05 and informed the effect sizes from our previously published study using this cohort (Li et al 2019). In this study we reported medium effect sizes using Cohen's (1988) criteria, ranging between 0.3-0.6. We have erred on the side of caution and conducted our power analysis with effect size 0.3 and α = 0.05.

Two power analysis were conducted. The first was for the arm of the study investigating TBI patients, where N = 34. We conducted a second power analysis for the analysis that include both TBI patients and healthy controls, N = 55.

The first power analysis specified two groups (task vs “rest”) and three conditions, or measurements (anodal, cathodal, or sham stimulation) and resulted in P = 0.836.

The second power analysis specified the same two groups (task vs “rest”), but four conditions (anodal, cathodal, or sham stimulation, and FA) and resulted in P = 0.992.

With power ≥ .80 we are confident our sample size provided adequate power to address the aims of this study.

**SUPPLEMENTARY INFORMATION:**

| **Participant ID** | **Report Summary** |
| --- | --- |
| TBI_01 | Mature damage and haemosiderin staining in L SFG, L MFG, L inferior frontal lobe, anterior temporal pole and posterior aspect L frontal operculum, R parieto-occipital and posterior temporal regions. Microhaemorrhages in b/l frontal border zone, dorsal pons. |
| TBI_02 | Mature parafalcine gliotic damage, L more than R. L temporal microhaemorrhages. |
| TBI_03 | Mature contusions b/l frontal poles, L lateral MTG. Microhaemorrhages L perisylvian cortex. |
| TBI_04 | Mature parafalcine contusions with L postcentral gyrus contusions, haemosiderin and damage of L thalamus. Subcortical microhaemorrhages bilaterally. |
| TBI_05 | Wallerian degeneration in the corticspinal tract. Evidence of petechial microhaemorrhages particularly in the right temporal lobe, L medial temporal lobe and left dorsal lentiform nucleus. |
| TBI_06 | Mature contusions with haemosiderin L frontal and parietal regions. Microhaemorrhages in R superior cerebellar pontine peduncle, R medial occipitotemporal gyrus, multiple perisylvian microhaemorrhages particularly on L. |
| TBI_07 | Small area of superficial siderosis in R SFG. |
| TBI_08 | Small contusion L occipital pole, some R pontine and L hemisphere atrophy. |
| TBI_09 | No mature contusions or microhaemorrhages |
| TBI_10 | R temporal contusions, R fronto-parietal-temporal superficial siderosis, parafalcine microhaemorrhages. |
| TBI_11 | R frontal and temporal pole superficial haemosiderin staining, superficial siderosis staining inferior surface R cerebellar |
| TBI_12 | Multiple subcortical microhaemorrhages, superficial siderosis R parietal and mature haemorrhagic contusions anterior frontal |
| TBI_13 | Bifrontal mature contusions, signal change at R parieto-occipital fissure possibly consistent with shear injury. |
| TBI_14 | R dorslolateral frontal and lateral temporal mature contusion |

| TBI_15 | L temporal and inferior frontal contusional gliotic damage, L parietal microhaemorrhage, extensive L superficial siderosis |
| --- | --- |
| TBI_16 | Evidence of some volume loss in the right cerebral peduncle and right hemi pons. |
| TBI_17 | Tiny T2* lesions in R anterior MFG. |
| TBI_18 | Minor parafalcine haemosiderin deposition to the right of the midline close the vertex in the superior frontal gyrus on SWI, and minor contusion in the left middle and inferior temporal gyri. |
| TBI_19 | L frontal sclerotic damage with haemosiderin. |
| TBI_20 | Non-specific subcortical WM spots. |
| TBI_21 | B/L mature inferior frontal pole and temporal pole contusions, few microhaemorrhages R MTL, L temporal pole. |
| TBI_22 | Few microhaemorrhages in L MTL. |
| TBI_23 | Microhaemorrhages parafalcine (R mostly), superficial haemosiderin deposition. |
| TBI_24 | Widespread microhaemorrhages. |
| TBI_25 | Mature haemorrhagic contusions R occipitotemporal, L lateral temporal, L dorsolateral frontal. Subcortical microhaemorrhages, especially R occipitotemporal. |
| TBI_26 | Scattered microhaemorrhages |
| TBI_27 | Mature R temporal and subfrontal contusion, L frontal microhaemorrhage |
| TBI_28 | Central sulcus superficial siderosis |
| TBI_29 | Microhaemorrhages L frontal parafalcine, B/L temporal, brainstem. Mature gliotic contusion R occipital. |
| TBI_30 | Microhaemorrhages L temporal pole, R SFG. Previous L cerebral peduncle haemorrhagic. |
| TBI_31 | Multiple microhaemorrhages frontal and deep white matter. Frontal pole signal change. |
| TBI_32 | Extensive microhaemorrhages R temporal, L cerebral peduncle. L SFG superficial siderosis. Perivenular haemorrhages R SFG and L MFG. Mature SFG & R supraorbital frontal contusions. |
| TBI_33 | Widespread microhaemorrhages. Mature R frontal contusions. |

***Table 1: summary of TBI patient structural MRI images***

Reports were based on T1, FLAIR and SWI/T2*GRE images.

Abbreviations: MFG=middle frontal gyrus, SFG=superior frontal gyrus, MTG=middle temporal gyrus, L=left, R=right, b/l=bilateral
